# Cross-talk between primary osteocytes and bone marrow macrophages for osteoclastogenesis upon collagen treatment

**DOI:** 10.1038/s41598-018-23532-x

**Published:** 2018-03-28

**Authors:** Jeevithan Elango, Christelle Sanchez, José Eduardo Maté Sánchez de Val, Yves Henrotin, Shujun Wang, Keolebogile Shirley Caroline Mamotswere Motaung, Ruihua Guo, Chunxiao Wang, Jeyashakila Robinson, Joe M. Regenstein, Bin Bao, Wenhui Wu

**Affiliations:** 1 Department of Marine Bio-Pharmacology, College of Food Science and Technology, Shanghai Ocean University, Shanghai, 201306 China; 2 Bone and Cartilage Research Unit, Arthropôle Liège, University of Liège, CHU Sart-Tilman, Liège, 4000 Belgium; 3 Department of Biomaterials Engineering, Universidad Católica San Antonio de Murcia, Murcia, Spain; 4 Co-Innovation Center of Jiangsu Marine Bio-industry Technology, Huaihai Institute of Technology, Lianyungang, 222005 China; 5 Department of Biomedical Sciences, TshwaneUniversity of Technology, Pretoria, 0001 South Africa; 6 Department of Fish Quality Assurance and Management, Fish Quality Monitoring and Certification Centre, Fisheries College and Research Institute, Tamil Nadu Fisheries University, Tuticorin, 628 008 India; 7 Department of Food Science, Cornell University, Ithaca, NY 14853–7201 USA

## Abstract

Homeostasis of osteoclast formation from bone marrow macrophages (BMM) is regulated by paracrine signals of the neighbourhood bone cells particularly mesenchymal stem cells (MSC), osteoblasts and osteocytes (OC). Besides paracrine cues, collagen and glycosaminoglycan are involved in controlling bone homeostasis. Towards this approach, different molecular weight collagens were reacted with MSC, OC and BMM to understand the bone homeostasis activity of collagen. The up-regulating effect of collagens on osteogenic cell growth was confirmed by the presence of mineralized nodules in the osteoblastogenic lineage cells and increased osteogenic stimulatory gene expression. The decreased BMM-derived TRAP+ osteoclasts number and osteoclastogenic regulatory gene expression of OC could demonstrate the exploitive osteoclastogenic activity of collagens. Osteoclastogenesis from BMM was triggered by paracrine cues of OC in some extend, but it was down-regulated by collagen. Overall, the effect of collagen on osteoclastogenesis and osteoblastogenesis may depend on the molecular weight of collagens, and collagen suppresses osteoclastogenesis, at least in part by downregulating the secretion of cytokines in OC.

## Introduction

Bone is connective tissue that continuously undergoes remodeling through the precise and localized coupling of resorption (removal of aged material) with replacement by newly formed bone. This process (bone homeostasis) requires regulated interactions between different cell types such as osteoblasts, osteoclasts (large plurinucleate cells), bone marrow macrophages and osteocytes. The failure of this system may help explain osteoporotic fractures in post-menopausal women and the elderly, a problem that has been increasing.

Osteoclast differentiation is triggered by cell-cell contact between osteoclast-precursor cells and other osteogenic progenitor cells in the bone including osteoblasts, osteocytes and bone marrow stromal cells^1^. The receptor activator of nuclear factor-kB ligands (RANKL) has been identified as the membrane-bound osteoclast differentiation factor of osteoclastogenesis-supporting cells that form stromal osteoclasts. Osteoblasts are believed to be the major cell type that expresses RANKL^2^. Nakashima *et al*.^3^ on the other hand stated that osteocytes have a greater capacity to support osteoclastogenesis *in vitro* than osteoblasts and bone marrow stromal cells. However, the major source of osteoclastogenesis remains unclear, as RANKL is expressed by several cell types in bone and bone marrow, including osteoblasts, osteocytes, mouse bone marrow mesenchymal stem cells (MMSC-bm) and lymphocytes. Therefore, it is important to know the actual cellular mechanisms of osteoclast formation to support work on bone remodeling.

Bone strength depends not only on the quantity of bone tissue but also on the quality, which is characterized by the geometry and the shape of bones, its mineral content and composition, and the fibrillar collagen microstructure^4^. Collagen is a potential candidate for bone tissue engineering and may provide innate biological guidance to cells that favor cell attachment and promote chemotactic responses. As an extracellular matrix protein, collagen plays an important role in bone strength and remodeling. During bone homeostasis, osteoblast cells undergo mineralization mainly within the bone collagen to form mature bone cells (osteocytes). It was reported that administration of porcine skin collagen hydrolysates enhanced bone growth^5^. In addition, oral administration of hydrolyzed type I collagen with calcitonin might inhibit bone resorption better than calcitonin alone^6^. Guillermin *et al*.^7^ reported that specific collagen amino acid sequences such as Asp-Gly-Glu-Ala, Ala, Hyp-, Gly-Pro-Val and Gly-Pro-Hyp are involved in bone metabolism and bone growth. Unlike type I collagen, the osteogenic mechanism of type II collagen (CII) has not been fully evaluated. Our previous study claimed the excellent biocompatible properties of shark type II collagen^8^. However, the influence of different molecular weight of type II collagens from shark cartilage with respect to the paracrine signaling mechanism of osteocytes for bone marrow macrophages derived osteoclast formation is needed to better understand for bone homeostasis and remodeling.

## Results and Discussion

### Effect of collagen on osteocytes

**Figure 1:**
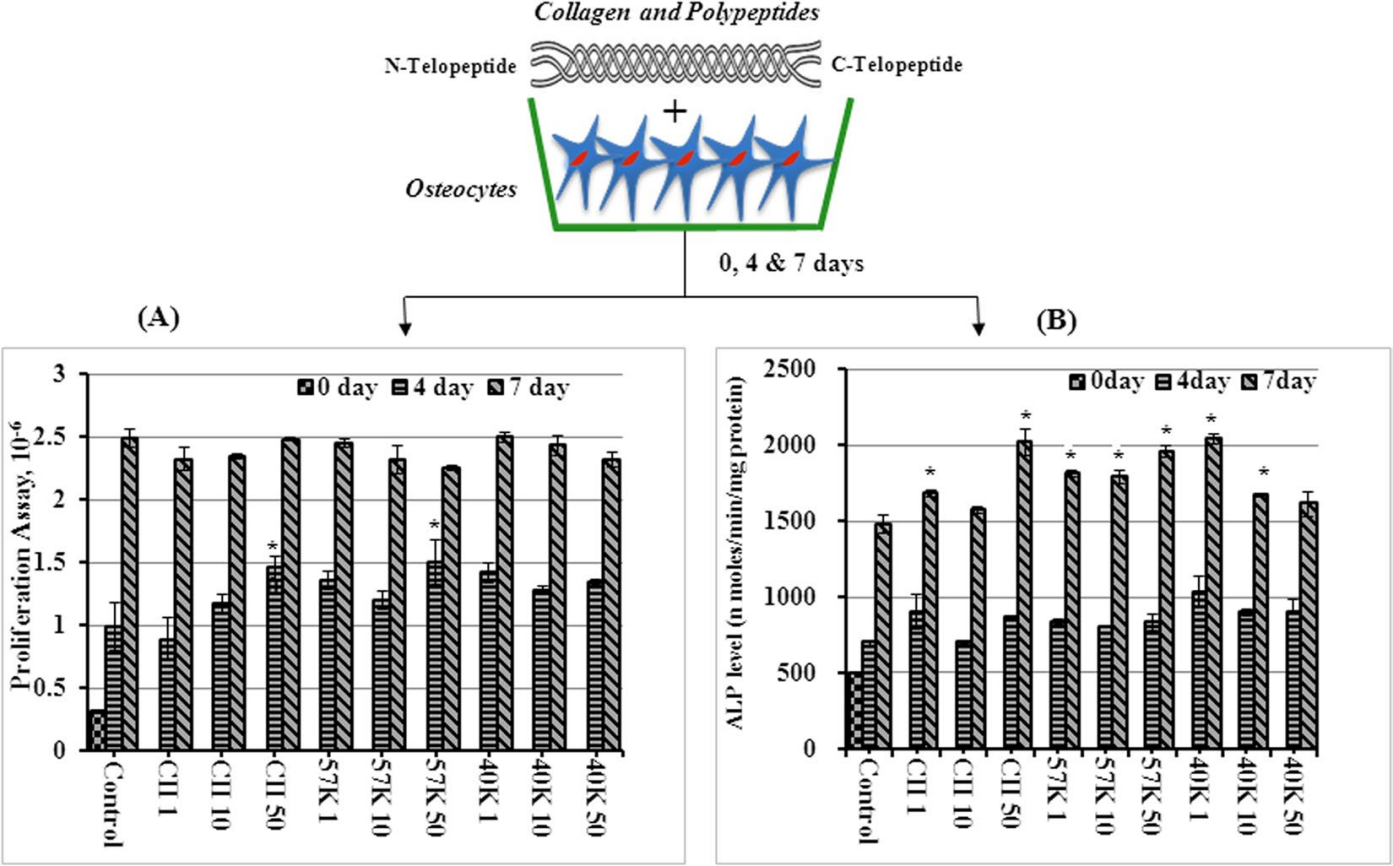
Proliferation effect (**A**) and ALP level (**B**) of pOC cells upon collagen and its polypeptides treatment. CII- type II collagen, 57 K and 40 K–57 and 40 kDa molecular weight collagen polypeptide, respectively. Data are from experiment repeated thrice with similar results. The values are mean + SEM (n = 3). *P < 0.05, vs. control.

The result of proliferation assay is given in Fig. 1. On day 4, type II collagen (CII) and 57 kDa type II collagen polypeptide (57 kDa) treated groups (50 μg/ml) had a greater number of osteocytes than control, however, there were no significant differences observed between treated and control group on day 7. Similarly, rat tail type I collagen (150 μg/ml) coated osteocyte-like cell lines (MLO-Y4 cells) found to be an effective way to maintain the growth of osteocyte-like phenotype^9^. In another study, primary osteocytes culture condition was optimized using type I collagen matrix with 0.2% FBS^10^.

In general, osteocytes would not produce a high level of ALP, therefore, as an exploratory study, we estimated ALP activity to understand the regulatory mechanism of collagen and polypeptides in osteocytes. The results showed that the ALP level was elevated more in CII (50 μg/ml), 40 kDa type II collagen polypeptide (40 kDa) (1 μg/ml) and 57 kDa polypeptide treated groups than control on day 7 (p < 0.05) (Fig. 1). On day 4, there were no changes on ALP level in the treated groups. ALP activity is considered as an important factor in determining bone cell differentiation and mineralization and is used as a biochemical marker for determining mature bone cell phenotype. Therefore, an increased level of ALP in osteogenic cells would indicate increased osteocytes activity. As shown in Fig. 1, ALP activity was elevated with increasing concentrations of collagen and its polypeptides from 1 to 50 μg/ml compared to control. Similar to the present findings, ALP activity of murine osteoblasts was significantly increased in the presence of 1 mg/mL porcine collagen hydrolysates^7^. Collectively, the above findings support an osteogenic capability of type-II collagens and also osteocytes might secrete ALP in some extent.

### mRNA expression

**Figure 2:**
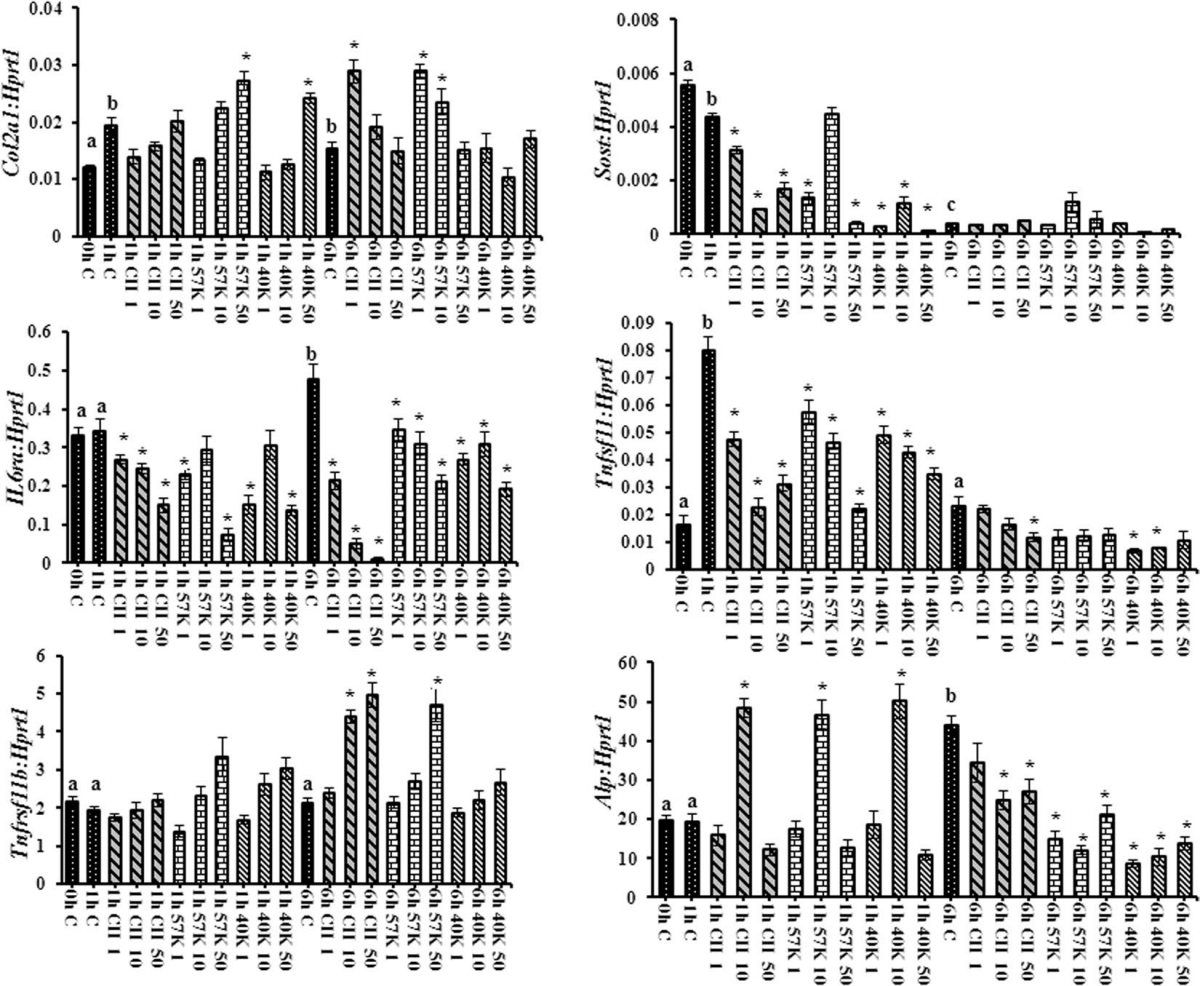
Expression of osteogenesis regulatory mRNA relative to *Hprt1* of osteocyte cells by real-time PCR. CII- type II collagen, 57 K and 40 K–57 and 40 kDa molecular weight collagen polypeptide, respectively. 1 h and 6 h represent a different time interval. Data are from experiment repeated thrice with similar results, *P < 0.05, vs. control. The values are mean + SEM (n = 3). Bars with different alphabets (a, b and c) are significantly different (P < 0.05) among controls at the different time point (0, 1 and 6 h).

mRNA levels of genes of interest in osteocytes treated with collagen and polypeptides are shown in Fig. 2. *Col2a1* mRNA levels were not significantly altered by either collagen or polypeptides treatment, except high dose (50 μg/ml) of polypeptides at 1 h treatment (p < 0.05) and, low dose of CII (1 μg/ml) and 57 kDa (1 and 10 μg/ml) had increased the level of *Col2a1* mRNA at 6 h. *Il6ra* mRNA levels were increased between 1 h and 6 h in untreated wells (p < 0.05). At 1 h and 6 h, *Il6ra* mRNA levels decreased with increasing CII and 57 kDa (50 μg/ml) (p < 0.05). It was proved that local stimulatory factors *IL-6* and *IL-6ra* had predictive for osteoclast maturation, activation, and recruitment. Several studies demonstrated the higher level of *IL-6ra* responsible for bone loss in early post-menopause^11,12^.

Tumor necrosis factor receptor superfamily member 11B (*Tnfrsf11b*) mRNA levels were not significantly altered by either collagen or polypeptide treatment in 1 h. At 6 h, *Tnfrsf11b* mRNA levels were increased with increasing collagen (10 and 50 μg/ml) and 57 kDa (50 μg/ml) (p < 0.05). There was no significant difference in *Tnfrsf11b* mRNA levels between controls and collagen polypeptides (1 μg/ml and 10 μg/ml)-treated cells. *Tnfrsf11b* gene provides instruction for making a protein osteoprotegerin, which plays a vital role in bone remodeling and regulation of osteoclast cells. *Tnfrsf11b* acts as a decoy receptor for the receptor activator of nuclear factor kappa B ligand (RANKL), a major cytokine for osteoclastogenesis^13^. Therefore, higher expression of *Tnfrsf11b* mRNA level by collagen and 57 kDa should reduce the osteoclast formation through osteocytes signaling mechanism.

Tumor necrosis factor ligand superfamily member 11 (*Tnfsf11*), also known as RANKL is a type-II membrane protein and has been identified to affect the immune system and control bone regeneration and remodeling. At 1 h and 6 h, *Tnfsf11* mRNA levels were decreased with increasing collagen and polypeptides (57 kDa and 40 kDa) concentration (50 μg/ml) (p < 0.05). There is strong evidence that osteocytes produce paracrine signals and regulate osteoclast formation through RANKL production^3^. The present study clearly depicted that treatment of collagen had obstructed the mRNA expression of RANKL in osteocytes.

*Sost* mRNA provides an instruction for producing a protein called sclerostin. It is produced in osteocytes and acts on the bone to stop bone formation. At 1 h, *Sost* mRNA levels were decreased with increasing collagen and polypeptides concentration (50 μg/ml) when compared to control cells (p < 0.05). There was no significant difference in *Sost* mRNA levels by collagen and polypeptides in 6 h. From the data, collagen had down-regulate bone formation inhibitor gene, i.e., *sost* mRNA expression, which further supported the proliferation effect of collagen and polypeptides on osteocytes (Fig. 1). The recent study reported the decreased formation of the mineralized matrix and an increased mRNA expression of *Sost* in osteocytes, indicating a reduction in bone formation via the NF-κB pathway^14^.

*Alpl* is the gene for the alkaline phosphatase (ALP) enzyme, which helps in the growth and development of bones through bone mineralization. This enzyme helps for the deposition of calcium and phosphorus in developing bones^15^. *Alpl* mRNA levels were increased significantly between 1 h and 6 h in untreated cells (p < 0.05). Except for CII, 57 kDa and 40 kDa (10 μg/ml)-treated cells, there was no significant difference in *Alpl* mRNA levels between controls and collagen/polypeptides-treated cells in 1 h. At 6 h, *Alpl* mRNA levels decreased in collagen/polypeptides-treated cells (p < 0.05). Though, the proliferation rate and cellular alkaline phosphatase level of collagen treated osteocytes increased, the level of *Alpl* mRNA expression was not triggered at 6 h treatment. Several hypotheses could be possible for the above result because in general, osteocytes are not the major sources for ALP productions for bone formation. The earlier study could demonstrate that an induction of *Alpl* mRNA during the *in vitro* osteogenic differentiation process expected for bone forming capacity of the bone marrow stromal cells *in vivo*^16^.

Taken together, these findings suggest that the expression of osteogenic regulatory genes such as *Col2a1* and *Alpl*, was triggered initially at 1 h and declined later at 6 h in collagens treated osteocytes; and osteoclasts regulatory genes, such as *Il6ra*, and *Tnfsf11* were suppressed in collagen and polypeptides treated osteocytes that could predict to down-regulate osteocytes cues for osteoclast formation. It was earlier confirmed by few authors that certain specific amino acid residues (asparagine, glutamine, glycine, and alanine) of collagen interact with integrin α_2_β_1_ on the mesenchymal stem cell membrane and can lead to up-regulate bone matrix synthesis through inhibition of TGF-beta signaling and activation of RUNX2 through FAK-JNK signaling^7,17,18^.

### Effect of collagen on MMSC-bm

**Figure 3:**
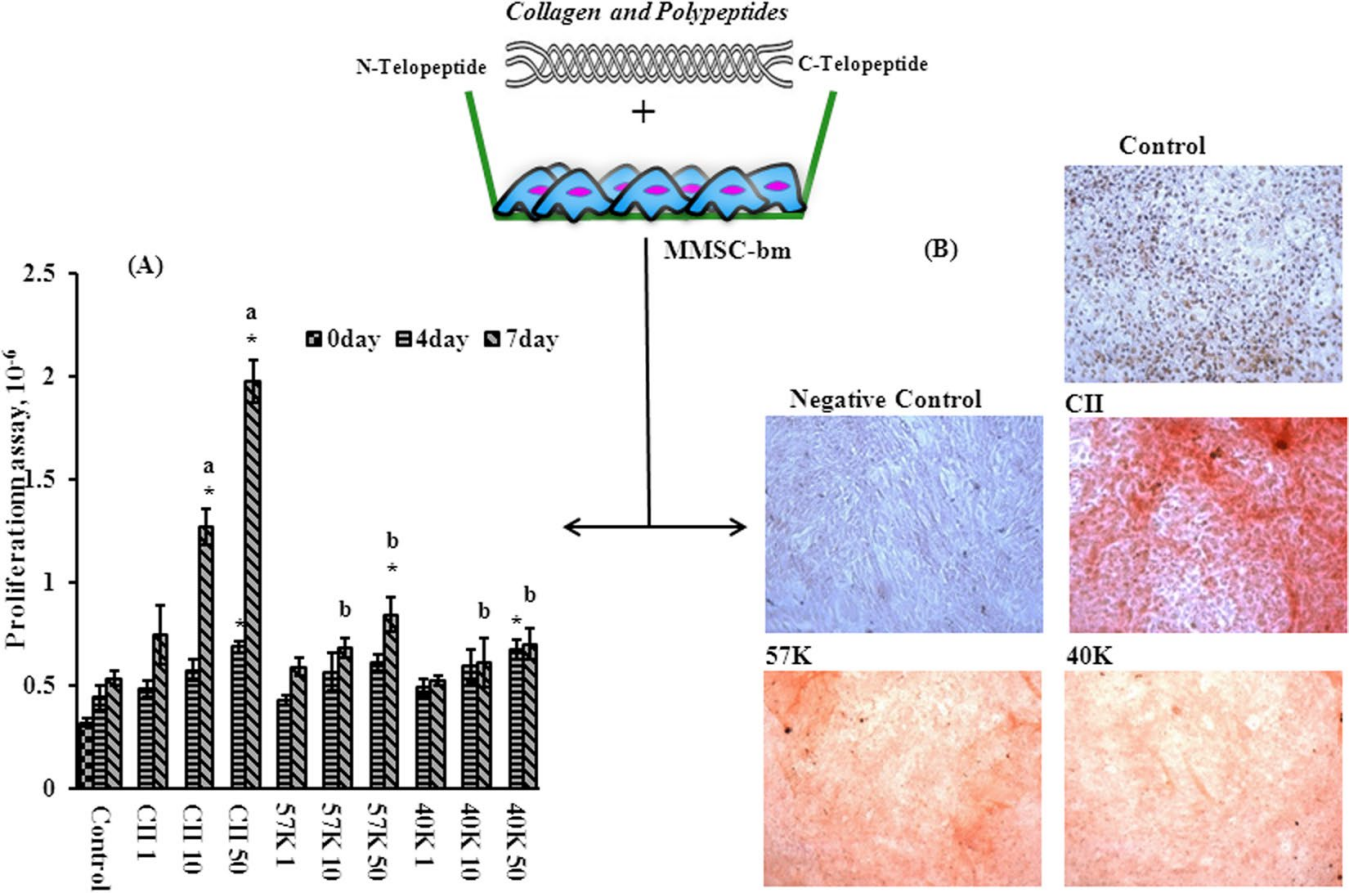
(**A**) Proliferation effect of collagen and its polypeptides on MMSC-bm cells. MMSC-bm cells cultured with collagen and polypeptides in the concentration of 1, 10 and 50 μg/ml. (**B**) Level of calcium deposit in MMSC-bm cells treated with collagen and polypeptides (50 μg/ml). Collagen treated MMSC-bm cells cultured with osteoblast medium containing osteoblast supplements for 21 days and stained with Alizarin red. Control and Negative control cells were cultured in osteoblastic medium with and without osteoblast supplements, respectively. CII- type II collagen, 57 K and 40 K–57 and 40 kDa molecular weight collagen polypeptide, respectively. Bars with different alphabets (a and b) are significantly different (P < 0.05) among CII and its polypeptides in their respective concentration. *P < 0.05, vs. control (MMSC-bm cells without collagen).

MMSC-bm activity contains three important phases including proliferation, matrix protein synthesis and mineralization of the bone matrix during new bone formation. The result of proliferation assay of MMSC-bm cells treated with different concentration of collagen and polypeptides is given in Fig. 3. In general, the proliferation rate of MMSC-bm cells was accelerated with increasing concentration of collagen and polypeptides. Notably, the rate was significantly higher on day 7 at 50 μg/ml of CII than other treatments. Similarly, Hennessy *et al*. and Gao *et al*. disclosed the osteogenic stimulatory activities of type I collagen peptides and rat tail type I collagen on mesenchymal stem cell^19,20^. In contrary, Song *et al*. observed decreasing trend of human bone marrow mesenchymal stem cells with coating and/or direct treatment of human type I collagen (5 μg/ml)^21^. Vleggeert-Lankamp *et al*. reported that bovine dermis type I collagen did not promote/inhibit the proliferation of human Schwann cells^22^. In some studies, type I collagen was shown having the effect to increase cell proliferation, whereas, in some others, it did not have such effect, even decreased the proliferation of MSC cells. We speculated that this could be due to different molecular composition of collagens from different sources. Recently, Chiu *et al*.^17^ reported that the level of integrin α_2_β_1_ complex (VLA-2) expression on MSC-bm surface increased by type-II collagen treatment on day 4 and then gradually reduced from day 6 to a lower level than that of the control on day 10.

Collagens and polypeptides treated MMSC-bm cells cultured in osteogenic media were able to differentiate into the osteoblastogenic lineage cells. This was confirmed by the presence of mineralized nodules in collagens treated MMSC-bm cells after Alizarin red staining on day 21 (Fig. 3). In collagens untreated cultures smaller aggregates were observed. There was no nodular aggregate deposited in negative control cultures (cells cultured without osteoblast supplements). This was clearly demonstrating that collagens and polypeptides accelerated osteoblast lineage cells differentiation from MMSC-bm and nodular aggregates present in collagens treated cultures were calcium deposits^23^. This is consistent with previous work showing that bone marrow-derived mesenchymal stem cells (BM-MSC) cultured on mammalian type-II collagen coated plates exhibited significant higher calcium deposition on day 12 and day 16, which suggests osteogenic induction properties of type-II collagen^17^. Collagen treated cultures had larger alizarin red positive aggregates and stained more intensively, indicating that a more extensive calcium deposition had occurred. The presence of alizarin red stain with nodular cell aggregates observed in collagen and polypeptides treated cultures establishes that these amorphous deposits contain calcium and suggests that calcium deposits are made up of MMSC-bm cells differentiated to the osteoblastic lineage. Numerous possible mechanisms have been proposed for stimulating MMSC-bm osteogenic differentiation^24,25^. Actual signaling mechanism of type II collagen during early osteogenic differentiation of MMSC-bm occurred by facilitating RUNX2 phosphorylation activation through integrin α_2_β_1_-FAK-JNK signaling axis, thus enhanced bone defect repair through an endochondral ossification-like process^17^.

### Effect of collagen on osteoclast differentiation

**Figure 4:**
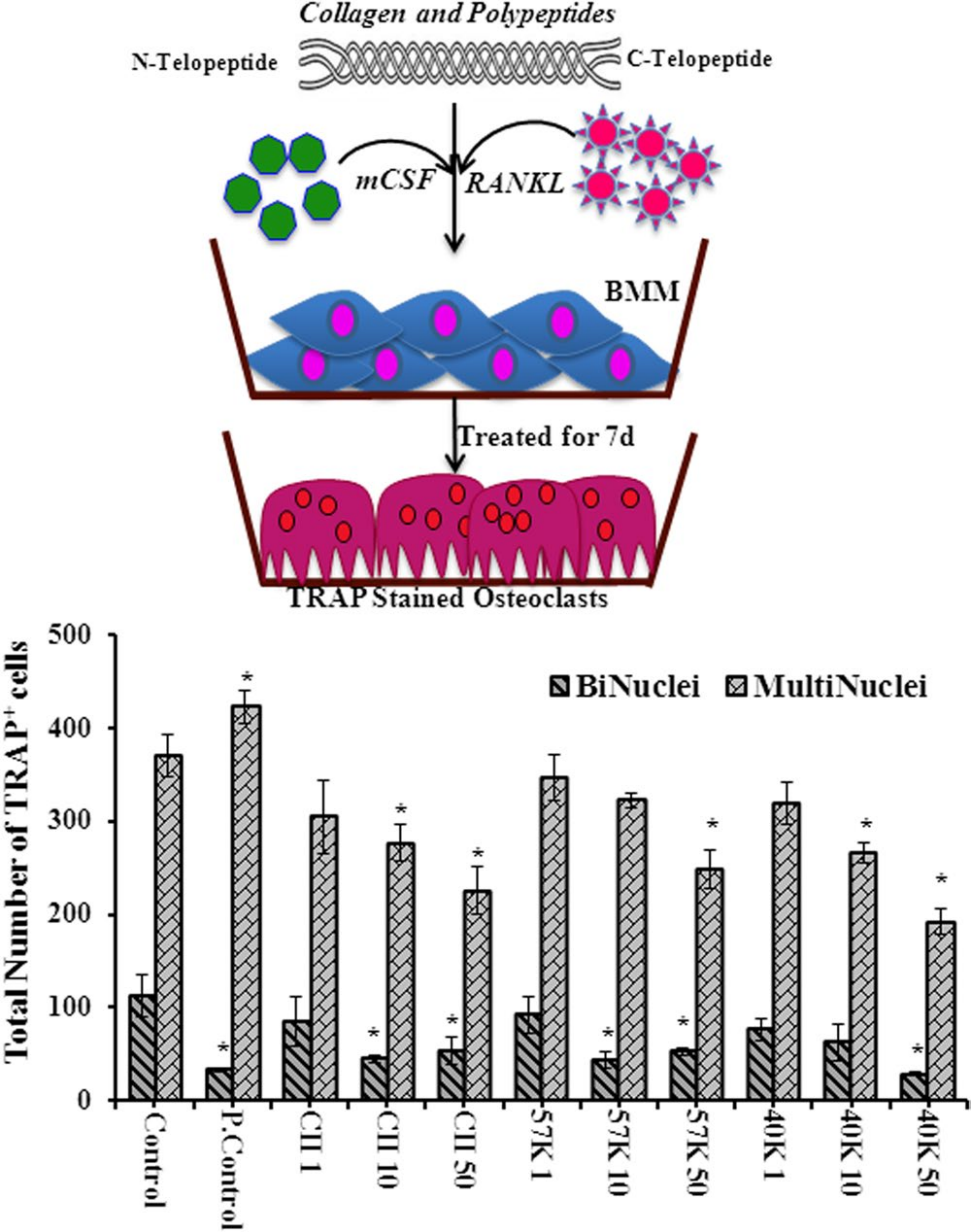
Effect of collagen and polypeptides on osteoclastogenesis. Control-cells treated with mCSF and RANKL alone, P. control (Positive control)-cells treated with oncostatin M-mCSF-RANKL, CII- type II collagen-mCSF-RANKL, 57 K and 40 K–57 and 40 kDa molecular weight collagen polypeptides-mCSF-RANKL, respectively. *p < 0.05 vs control.

Macrophages harvested from mouse bone marrow were treated with RANKL and mCSF in the presence and absence of different concentration of collagen and polypeptides to assess the effects of the collagens on osteoclastogenesis. The result shows that increasing concentration of collagen and its polypeptides reduced osteoclast numbers considerably (p < 0.01) (Fig. 4). In contrast, the murine oncostatin M (m-OSM) treated group increased osteoclast cell numbers (3+ nuclei) compared to control. But, the number of double nuclei osteoclast cells was decreased in m-OSM treated group than control (p < 0.01). The higher concentration of collagen and polypeptides treated groups had considerably decreased both double nuclei and 3+ nuclei TRAP+ cells compared to control (p < 0.01). Similarly, Guillermin *et al*.^7^ reported that murine osteoclasts differentiation reduced by the treatment of porcine collagen hydrolysates (2 or 5 kDa) with 1 mg/ml concentration, which might be due to inhibition of Transforming growth factor beta (TGF-β) through interaction of collagen-derived peptides (asparagine, glycine, glutamine and alanine).

**Figure 5:**
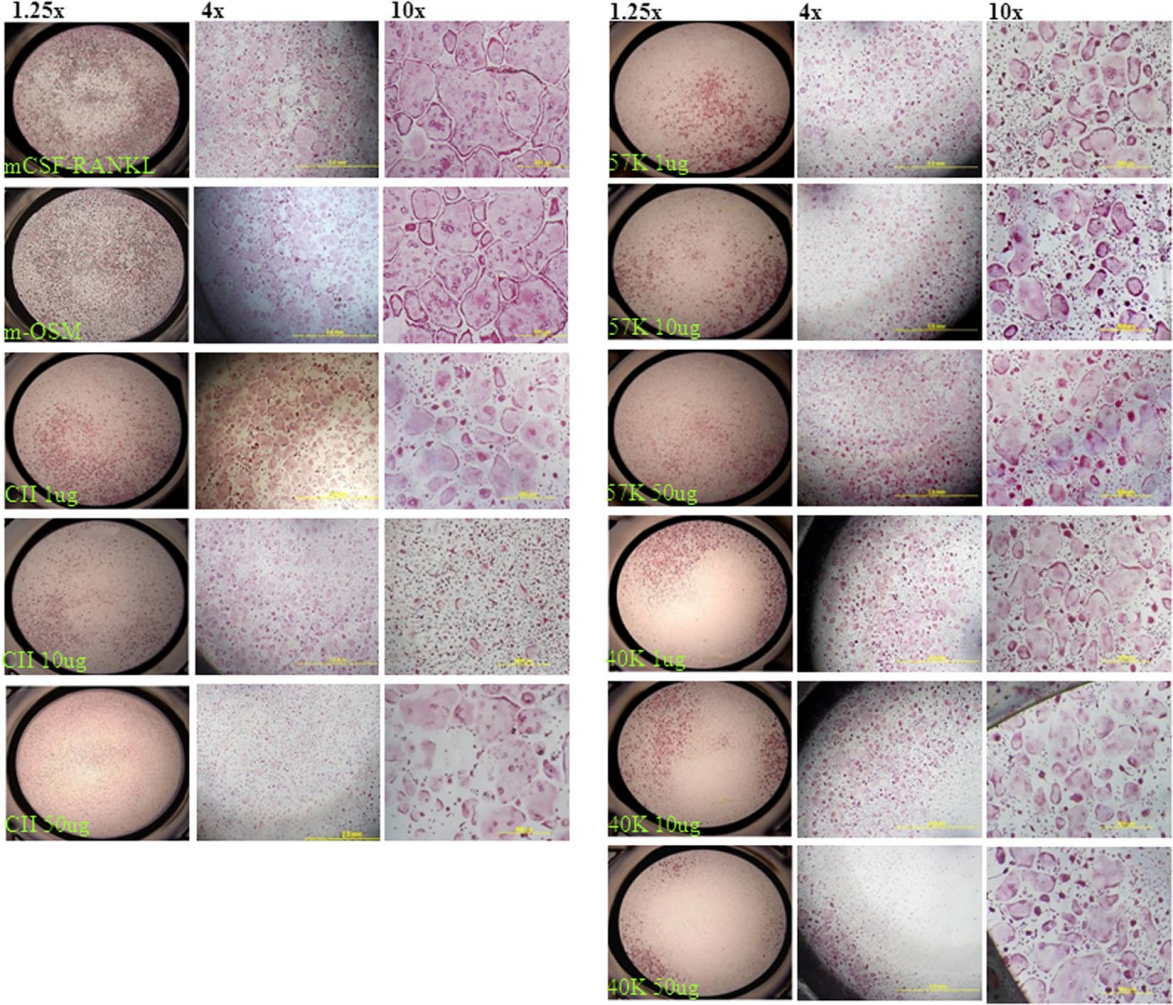
Total number of TRAP+ osteoclast cells (double nuclei and three plus nuclei) between control and collagen treatment. OSM-oncostatin-mCSF-RANKL, M CII- type II collagen-mCSF-RANKL, 57 K and 40 K–57 and 40 kDa molecular weight collagen polypeptides-mCSF-RANKL, respectively. Data are from experiment repeated thrice with similar results (*p < 0.05). The values are mean + SEM (n = 3).

The micrograph clearly depicted the lower number of osteoclast cells in the presence of higher concentration (50 μg/ml) of collagen and its polypeptides (Fig. 5). Among the treatment, 1 μg of 57 kDa collagen polypeptide-treated group had a high number of osteoclast cells than other treatment groups. In higher magnification (10×), “ghost” outlines remaining after osteoclast apoptosis cells were found in wells treated with the higher doses of CII but not in lower dose treatment. This observation indicated that increasing concentration of collagen increased apoptosis in osteoclasts.

### Co-culture model

**Figure 6:**
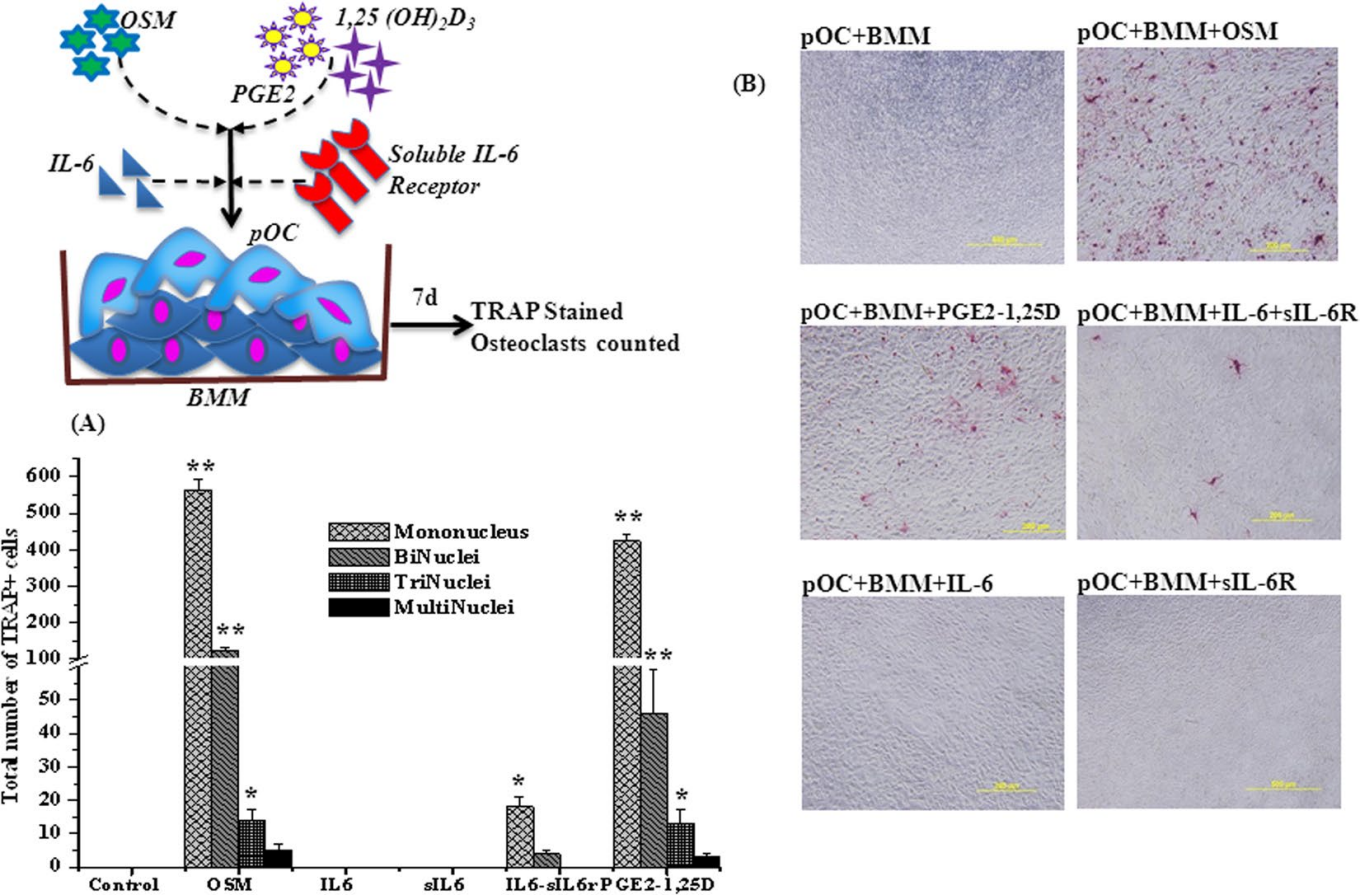
(**A**) Effect of Inducers on osteoclast differentiation by co-culture of bone marrow macrophages with p-osteocytes for 7 days. (**B**). Microscopic structure of BMM co-cultured with p-osteocytes in presence of inducers. OSM-oncostatin M, IL6- Interleukin 6, sIL6R- soluble interleukin 6 receptor, PGE2-prostaglandin E_2_, 1,25D- 1α,25-Dihydroxyvitamin D3. (*p < 0.05; **p < 0.001 vs control). The values are mean + SEM (n = 3).

It was reported that differentiated primary osteoblasts express cytokines, RANKL and mCSF in presence of stimulators (IL-6, soluble IL-6 Receptor, prostaglandin E2 (PGE2), 1,25-Dihydroxy vitamin D3 (1,25 (OH)_2_D_3_) and oncostatin M), which bind with mouse BMM and support osteoclast differentiation^24^. In contrast, Nakashima *et al*.^3^ identified that purified osteocytes also express a much higher amount of RANKL and have a greater capacity to support osteoclastogenesis. Therefore, in the present study, we used a co-culture model with different stimulators to understand the effect of inducers such as m-OSM, IL-6, sIL-6R, 1,25 (OH)_2_D_3_ and PGE2 on osteoclast formation. The primary co-culture of BMM with pOC was grown in presence of inducers for 7 days. m-OSM significantly increased the number of single and double nuclei TRAP-positive cells, but few cells with more than 2 nuclei were observed. Compared to m-OSM, less number of TRAP-positive cells found in 1,25 (OH)_2_D_3_ and PGE2 group. In contrast, IL-6 in the presence of sIL6R supported only the production of TRAP-positive single and double  nuclei cells to some extent (Fig. 6). No TRAP-positive cells were observed in wells treated with IL-6 or sIL-6R alone; this can also be seen in the micrographs (Fig. 6).

**Figure 7:**
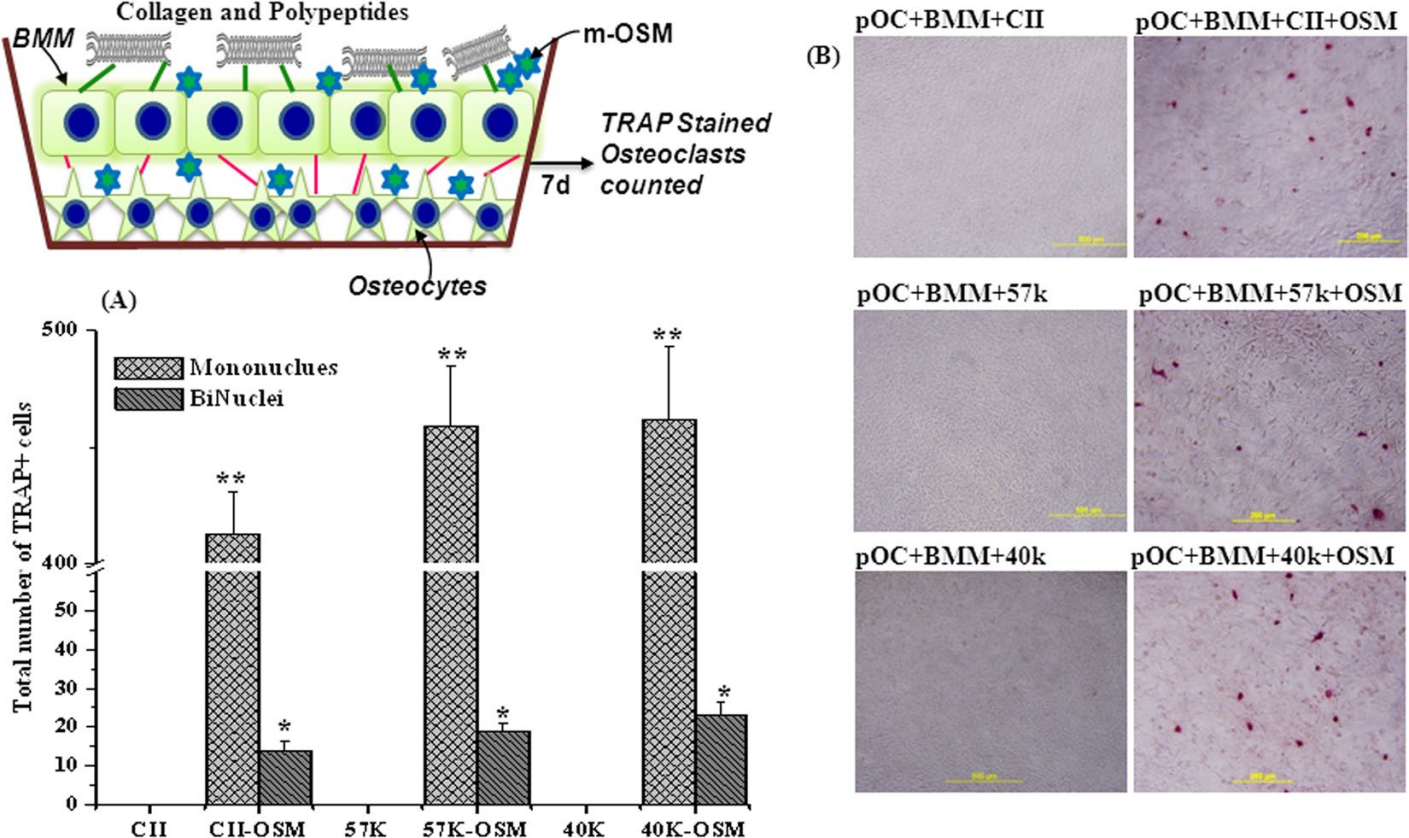
Formation of TRAP-positive cells (**A**) and microscopic structure (**B**) of bone marrow macrophages (BMM) co-cultured with p-osteocytes in presence of oncostatin M (m-OSM) with or without collagens. CII- type II collagen, 57 K and 40 K–57 and 40 kDa molecular weight collagen polypeptides, respectively. Data are from experiment repeated thrice with similar results. *p < 0.05; **p < 0.001 vs collagen/polypeptide alone. The values are mean + SEM (n = 3).

Our above study confirmed that osteocytes stimulated with m-OSM might support osteoclast differentiation to the point of a multinucleated cell, as previously observed with other stimuli in freshly isolated osteocytes^26^. Further, we aimed to understand whether collagen and its polypeptide might play a similar role in the presence and absence of inducer, OSM in the co-culture of BMM and pOC. TRAP-positive single and double nuclei cells were formed in the collagens-OSM treated group (Fig. 7), but it was lower than m-OSM alone treated group (Fig. 6), which confirmed that collagen and collagen polypeptides might suppress m-OSM-stimulation of osteocytes towards osteoclast formation to some extent. In contrast, others reported no effect of hydrolyzed collagen on primary co-culture of osteoblast and osteoclasts growth^7^. The down-regulation of osteoclastogenesis by collagen might be justified by the higher level of Tnfrsf11b mRNA (a decoy receptor for RANKL) (Fig. 2) that coupled with m-OSM-triggered-RANKL and leads to inhibition of RANK-RANKL ligand binding towards osteoclast formation.

The level of inhibition was higher (p < 0.05) in a collagen-OSM group than polypeptides treated groups, which might be due to the suppressive mechanism of osteoclastogenic regulatory genes such as *Il6ra* and *Tnfsf11* expression of pOC by collagen (Fig. 2). Thus, pOC could not secrete necessary cytokines in order to stimulate osteoclast formation from collagen treated BMM-pOC co-culture, though the presence of positive inducer, m-OSM.

## Methods

### Materials

Type II collagen from whale shark cartilage was extracted with 0.5 M acetic acid containing 1% pepsin (Sigma-Aldrich Co., Ltd, Shanghai, China) and purified by gel-filtration column chromatography using a Sephadex G-100 (Sigma-Aldrich) column (25 × 3 cm). Collagen polypeptides were obtained by enzyme (thermolysin from Bacillus thermoproteolyticus rokko, EC number: 3.4.24.27, Sigma-Aldrich) hydrolysis of collagen^8^. Briefly, 100 mg/mL collagen in 50 mM sodium phosphate buffer (pH 7.0) was hydrolyzed with thermolysin at 0.1% (w/w) of the protein content, 70 °C) for 60 min and terminated with the addition of EDTA (20 mM) and two types of (57 and 40 kDa) collagen polypeptides were obtained using molecular weight cut-off filters (Millipore, Shanghai, China)^8^.

### Effect of collagen and collagen polypeptides on MMSC-bm and osteocytes

#### Cell culture

Primary osteocytes (pOC) cells were harvested from mice^27^ (Sino-British Sippr/BK Lab Animal Co., Ltd, Shanghai, China) long bones (femora, and tibia) using 300 U/mL collagenase (Sigma-Aldrich) dissolved in α-minimal essential medium (α-MEM) (Gibco, Shanghai, China) and were cultured in α-MEM medium supplemented with 10% fetal bovine serum (Gibco) at 37 °C in a CO_2_ incubator (Shanghai Hengyue Medical Instruments Co., Ltd, Shanghai, China), respectively. Animal study protocols and procedures were approved by the Shanghai Ocean University institutional animal care and use committee (Permit Number: 13-0012). All methods were employed in accordance with the relevant guidelines and regulations of Scientific and Ethical Care and Use of Laboratory Animals of Shanghai Ocean University.

Mouse bone marrow-mesenchymal stem cells (ZQ0465) (MMSC-bm) were purchased from the Shanghai Zhong Qiao Xin Zhou Biotechnology Co., Ltd, Shanghai, China and were cultured in mesenchymal stem cell culture medium (brand: Sciencell, Cat. No. 7501) containing 10% fetal bovine serum (FBS), (brand: Sciencell, Cat. No. 0025), mesenchymal stem cell growth supplement (1% MSCGS, Cat. No. 7552) and 1% penicillin/streptomycin (10,000 units/ml of Penicillin and 10,000 μg/ml of Streptomycin in a saline solution) (Cat. No. 0503) at 37 °C in a CO_2_ incubator.

#### Proliferation assay

After confluence, cells pOC and MMSC-bm at passage 5 were seeded (5 × 10^5^ cells/well/48 well plate) in microtiter plates (Costar, Shanghai) along with the different concentration of collagen and polypeptides (1, 10 and 50 μg/ml). Controls consisted of uncoated (without collagen) wells. The total number of viable cells was counted using an Invitrogen cell counter (Countess II Automated Cell Counter, ThermoFisher Scientific, Shanghai, China) at 0, 4 and 7 days after seeding.

#### Alkaline phosphatase (ALP) assay

pOC cells were generated as described above. At each time point, cells were washed twice with 1 × phosphate buffered saline (PBS), harvested with lysis buffer (10 mM Tris buffer, pH 7.4) and sonicated (40 w, Shanghai Kedao Ultrasonic Instrument Co, Shanghai, China) with an ice water-bath for 30 sec to release cellular components. After a brief centrifugation (13,000 × g for 1 min at 4 °C, Universal 320 R, Andreas-Hettich, Buckinghamshire, Germany), the supernatant was used for the ALP assay. In brief, 25 µl per sample was added to 100 µl substrate (10 mM p-nitrophenyl phosphate (Sigma-Aldrich) in assay buffer (0.1 M Na_2_CO_4_ buffer, pH 10.0)). The reaction proceeded for 30 min at 37 °C; the reaction was terminated by addition of 50 µl NaOH (1 M) and absorbance measured at 410 nm using a plate reader (Bio-Rad Model 550, Shanghai). ALP activity was determined from a standard curve using 10 mM p-nitrophenol from 4000 to 62.5 nmoles and without substrate maintained as a control. The same volume of sample was used to determine protein content using bicinchoninic acid (BCA) as per the manufacturer’s instructions (Pierce, IL, USA). ALP activity was expressed as nmoles/min/mg protein determined as follows:

$$\begin{array}{ll}{\rm{Alkaline}}\,{\rm{Phosphatase}}\,{\rm{concentration}}: & {\rm{ALP}}\,{\rm{level}}\times \mathrm{1,000}\\ (\text{nmoles}/\min /\text{mg}\,{\rm{protein}}) & \,\,{\rm{PC}}\times {\rm{Time}}\end{array}$$

where, ALP level- Alkaline phosphatase level (nmoles/ml), PC- Protein concentration (μg/ml),Time-30 min

#### mRNA expression

pOC cells were seeded (250,000 cells/well) in 6 well microtiter plates (Costar) along with the different concentration of collagen and polypeptides (1, 10 and 50 μg/ml). Controls consisted of uncoated (without samples) wells. The percentage of mRNA expression (*Sost*, *Alpl*, *Il6ra*, *Tnfsf11*, *Tnfrsf11b* and *Col2a1*) was determined at a different time interval (0, 1 and 6 h) as follows.

#### RNA Extractions (Trizol Method)

Culture medium was removed from the cells by pipetting after an incubation period and washed twice with ice-cold PBS. The cell monolayer was lysed in TRIzol (Invitrogen Life Technologies, Shanghai, China) (0.5 ml/well). The Trizol supernatant was passed through a 21 G needle using Luer-lock syringes (Zogear Industries Co.,Ltd, Shanghai, China) to shear the RNA. Phase separation was obtained by addition of 0.2 ml chloroform for each 1 ml of Trizol used and shaken vigorously by hand until the sample was opaque. Then cells were left on ice for a few min and spun at 12,000 × g for 15 min at 4 °C. The top clear phase containing RNA was collected and added to a fresh tube containing 0.5 ml isopropanol for RNA precipitation. The mixture was mixed thoroughly and centrifuged as previously. The pellet was washed with 0.5 ml 70% ethanol and re-centrifuged. The RNA pellet was suspended in an appropriate volume (10 μl) of RT-PCR water (Shanghai Biocolor BioScience & Technology Company, Shanghai, China). DNase treatment was done using a Turbo DNase kit (Shanghai Biocolor BioScience & Technology Company, Shanghai, China) as per the manufacturer’s instructions. After centrifuging at 1000 × g for 2 min at 4 °C, RNA was quantified at an absorbance of 260 nm using a Nanodrop ND-1000 spectrophotometer (ThermoFisher Scientific).

#### cDNA synthesis

First strand cDNA synthesis was done in a 20 μl reaction mixture containing 1.0 μg RNA, 10 μl FS master mix (Invitrogen, Shanghai, China), 3 μl random primer (Invitrogen), 1 μl affinity script (Invitrogen) and Molecular grade water to 20 μl. The reaction mixture was incubated at 42 °C for 30 min and 85 °C for 5 min, followed by 5 min at 90 °C to inactivate the RT in a Biometra T3000 thermocycler 48 (Biometra Gmbh, Gottingen, Germany).

Real-time polymerase chain reaction (RT-PCR) was done using a 96-well plate using an ABI 7500 Fast Real-Time PCR System (Applied Biosystems, Shanghai, China) using SYBR Green Fast qPCR RT Master Mix (Invitrogen, Shanghai, China). pOC regulatory primers designed using Primer-BLAST (Shanghai Biotechnology Co., Ltd, Shanghai, China) were listed in Table 1. The total volume of each PCR reaction was 10 μl, containing 5 μl SYBR Green Fast qPCR RT Master mix, 1.5 μl cDNA template sample, 0.5 μl of forward and reverse primers and 2.5 μl water. The PCR reaction was carried out at 95 °C for 30 min, 40 cycles at 95 °C for 5 min, 60 °C for 30 min, one cycle of 95 °C for 1 h, 55 °C for 30 min and 95 °C for 30 min.

**Table 1:** List of primer sequence used in this study.

Primers	Sequence
*Hprt1*	F′-TGATTAGCGATGATGAACCAG
(housekeeping gene)	R′-AGAGGGCCACAATGTGATG
*Sost*	F′-GAGAACAACCAGACCATGAAC
	R′-GCTCGCGGCAGCTGTACT
*Alpl*	F′AAACCCAGACACAAGCATTCC
	R′-TCCACCAGCAAGAAGAAGCC
*Tnfsf11*	F′-TCCAGCTATGATGGAAGGCT
	R′-GTACCAAGAGGACAGAGTG
*Tnfrsf11b*	F′CCTACCTAAAACAGCACTGCAC
	R′-TAACGCCCTTCCTCACACTC
*Il6ra*	F′-TGCAACGCCATCTGTGAGT
	R′-CTGGACTTGCTTCCCACACT
*Col2a1*	F′-ATCTGTGAAGACCCAGACTGC
	R′-GTTCTCCTTTCTGCCCCTTTG

#### Osteogenic differentiation and quantification

MMSC-bm cells at passage 5 were initially seeded at a density of 5 × 10^4^ cells/well in microtiter 6 well plates. After 2 h, 50 μg/ml collagen or collagen polypeptides were added into each well. To induce osteogenesis, the cells were grown in osteoblast medium (Shanghai Zhong Qiao Xin Zhou Biotechnology Co., Ltd, Cat. No. 4601) with the addition of osteoblast growth supplement (ObGS) (Shanghai Zhong Qiao Xin Zhou Biotechnology Co., Ltd, Cat. No. 4652) composed of 100 nM dexamethasone, 10 mM b-glycerolphosphate, and 0.05 mM 2-phosphate-ascorbic acid for 21 days with media changes every 3 days. Control and negative control cells were grown in the culture medium without sample and ObGS, respectively. The effect of collagen on osteogenesis was confirmed by observing Ca deposition in cultured cells using the Alizarin red staining method^28^. Briefly, cells were washed with PBS and fixed in 4% paraformaldehyde for 30 min and stained with 1% alizarin red (Sinopharm Chemical Reagent Co., Ltd, Shanghai, China) containing 1% NH_4_OH.

### Effect of collagens on osteoclastogenesis

#### Bone marrow macrophage (BMM) preparation and RANKL-induced osteoclastogenesis

Mouse bone marrow macrophages (BMM) from 8 wk old wild mice (Sino-British) were cultured in a T75 medium flask (Costar) (20 ml final volume) containing 30 ng/ml mCSF (R&D Industries, Minneapolis, MN, USA) in endotoxin-free RPMI-10% heat-inactivated fetal bovine serum (FBS) (Gibco). The cells were cultured for 3 days and non-adherent BMM precursors were collected by centrifugation at 1,500 g for 4 min at 4 °C. The cells were re-suspended in αMEM medium (Gibco) and cell counts were adjusted to 50 × 10^5^ BMM/ml. Then, they were seeded at 10^5^ cells/well/0.2 ml medium in 48 well plates and treated with RANKL (100 ng/ml)/mCSF (30 ng/ml) (R&D Industries) (control group), mouse oncostatin M (m-OSM)-RANKL-mCSF (positive control), collagen and polypeptides (1, 10 and 50 μg/ml)-RANKL-mCSF. The plates were incubated at 37 °C in a humidified atmosphere with 5% CO_2_ for 7 days. Cells were fixed in 4% formaldehyde (Sinopharm) for 3 min followed by ethanol: acetone (1:1) for 1 min. After brief air dry, the cells were incubated with tartrate-resistant acid phosphatase (TRAP) stain (Sigma) for 8 min^29^, and the number of double and triple plus nucleated TRAP-positive osteoclasts were counted under a light microscope (Olympus Inc.; Center Valley, PA, USA).

#### Co-culture model

BMM cultured as above were co-cultured with pOC (2 × 10^4^ cells/ml) in the presence of inducers such as oncostatin-M (m-OSM, 50 ng/ml), 10 nM 1α,25-Dihydroxyvitamin D3 (1, 25 (OH)_2_ D_3_), 100 nM prostaglandin E_2_, IL-6 (40 ng/ml) and soluble IL-6 receptor (100 ng/ml) (R&D Industries). BMM was also co-cultured with osteocytes in the presence of m-OSM (50 ng/ml) with or without collagen and polypeptides (50 μg/ml). From the previous results, m-OSM was selected as the potential inducer. Plates were incubated at 37 °C in a humidified atmosphere with 5% CO_2_ for 7 days, with a media change at day 3. Cells were then fixed with 4% paraformaldehyde (Sigma-Aldrich) for 10 min on ice, single and double plus nucleated osteoclasts were counted after TRAP stain.

## Conclusion

In summary, the *in-vitro* results obtained with pOC, MMSC-bm and osteoclast cells demonstrated that type-II collagen and its polypeptides were able to stimulate osteogenesis and supress osteoclastogenesis. The effect of collagen and its polypeptides in osteocytes and MMSC-bm cell growth is dose-dependent, which ultimately maintained the bone formation. Osteoclast numbers were reduced by the concentration of collagen and its polypeptides. Intriguingly, a higher concentration of type-II collagen and polypeptides treatment had up-regulated the osteoblast lineage cell differentiation from MMSC-bm and had down-regulated the BMM-derived osteoclast cell formation, suggesting that collagen or collagen hydrolysate could be helpful in the management of bone diseases like osteoporosis. There are numerous reports disclosing the chondrogenic properties of type II collagen for cartilage repair, but here we are reporting that not only chondrogenesis, type II collagen might also play some role in ossification through modulating osteocytes paracrine signals for osteoclastogenesis. Therefore, the present work concluded that type II collagen and collagen polypeptides isolated from shark cartilages might be conceivable novel biomaterials to modulate bone formation and resorption activity; and could be of potential curiosity as a nutritional supplement in the preclusion of bone loss in osteoporosis. This work also highlighted that in some parameters native collagen was more efficient than that observed in collagen polypeptides. However, one important issue that needs to be addressed by further studies is the concern on the molecular interaction of collagen with osteocytes in order to maintain bone homeostasis.

### Data Availability

The datasets generated and analyzed during the present study are available from the corresponding author upon reasonable request.

## References

[1] Takahashi N (1988). Osteoblastic cells are involved in osteoclast formation. Endocrinol..

[2] Suda (1999). Modulation of osteoclast differentiation and function by the new members of the tumor necrosis factor receptor and ligand families. Endocrinol. Rev..

[3] Nakashima T (2011). Evidence of osteocyte regulation of bone homeostasis through RANKL expression. Nat Med..

[4] Viguet-Carrin S, Garnero P, Delmas PD (2006). The role of collagen in bone strength. Osteoporosis Int..

[5] Wu J, Fujioka M, Sugimoto K, Mu G, Ishimi Y (2004). Assessment of effectiveness of oral administration of collagen peptide on bone metabolism in growing and mature rats. J Bone Miner Metab..

[6] Adam M, Spacek P, Hulejová H, Galiánová A, Blahos J (1996). Postmenopausal osteoporosis. Treatment with calcitonin and a diet rich in collagen proteins. Cas Lek Cesk..

[7] Guillerminet F (2010). Hydrolyzed collagen improves bone metabolism and biomechanical parameters in ovariectomized mice: An *in vitro* and *in vivo* study. Bone..

[8] Jeevithan E (2016). Biocompatibility assessment of type-II collagen and its polypeptide for tissue engineering: effect of collagen’s molecular weight and glycoprotein content on tumor necrosis factor (Fas/Apo-1) receptor activation in human acute T-lymphocyte leukemia cell line. RSC Adv.

[9] Kato Y, Windle JJ, Koop BA, Mundy GR, Bonewald LF (1997). Establishment of an osteocyte-like cell line, MLO-Y4. J Bone Miner Res..

[10] Honma M, Ikebuchi Y, Kariya Y, Suzuki H (2015). Establishment of optimized *in vitro* assay methods for evaluating osteocyte functions. J Bone Miner Metab..

[11] Seck T, Diel I, Bismar H, Ziegler R, Pfeilschifter J (2002). Expression of interleukin-6 (IL-6) and IL-6 receptor mRNA in human bone samples from pre- and postmenopausal women. Bone.

[12] Scheidt-Nave C (2001). Serum interleukin-6 is a major predictor of bone loss in women specific to the first decade past menopause. J Clin Endocrinol Metab..

[13] Remuzgo-Martínez S (2016). Expression of osteoprotegerin and its legends, RANKL and TRAIL, in rheumatoid arthritis. Sci Rep..

[14] Kobayashi K (2015). Mitochondrial superoxide in osteocytes perturbs canalicular networks in the setting of age-related osteoporosis. Sci Rep..

[15] Bellows CG, Aubin JE, Heersche JN (1991). Initiation and progression of mineralization of bone nodules formed *in vitro*: the role of alkaline phosphatase and organic phosphate. Bone Mineral..

[16] Prins HJ (2014). *In vitro* induction of alkaline phosphatase levels predicts *in-vivo* bone forming capacity of human bone marrow stromal cells. Stem Cell Res..

[17] Chiu LH (2014). The effect of type II collagen on MSC osteogenic differentiation and bone defect repair. Biomaterial..

[18] Tuckwell DS, Ayad S, Grant ME, Takigawa M, Humphries MJ (1994). Conformation dependence of integrin-type II collagen binding Inability of collagen peptides to support alpha 2 beta 1 binding, and mediation of adhesion to denatured collagen by a novel alpha 5 beta 1-fibronectin bridge. J. Cell Sci..

[19] Hennessy KM (2009). The effect of collagen I mimetic peptides on mesenchymal stem cell adhesion and differentiation, and on bone formation at hydroxyapatite surfaces. Biomaterial..

[20] Gao C (2013). MSC-seeded dense collagen scaffolds with a bolus dose of VEGF promote healing of large bone defects. Eur. Cell. Mater..

[21] Song G, Ju Y, Soyama H (2008). Growth and proliferation of bone marrow mesenchymal stem cells affected by type I collagen, fibronectin and bFGF. Mater. Sci. Eng..

[22] Vleggeert-Lankamp CL (2004). Adhesion and proliferation of human Schwann cells on adhesive coatings. Biomaterial..

[23] Gregory CA, Gunn WG, Peister A, Prockop DJ (2004). An alizarin red-based assay of mineralization by adherent cells in culture: comparison with cetylpyridinium chloride extraction. Anal Biochem..

[24] Marie P, Debias F, Cohen-Solal M, de Vernejoul MC (2000). New factors controlling bone remodeling. Joint Bone Spine.

[25] Kartsogiannis V, Ng KW (2004). Cell lines and primary cell cultures in the study of bone cell biology. Molecul Cell Endocrinol..

[26] Zhao S, Zhang YK, Harris S, Ahuja SS, Bonewald LF (2002). MLO-Y4 osteocyte-like cells support osteoclast formation and activation. J Bone Miner Res..

[27] Stern AR (2012). Isolation and culture of primary osteocytes from the long bones of skeletally mature and aged mice. Biotechnique..

[28] Donzelli E (2007). Mesenchymal stem cells cultured on a collagen scaffold: *In vitro* osteogenic differentiation. Arch Oral Biol..

[29] Parkinson IH, Fazzalari NL, Durbridge TC, Moore AJ (1991). Simplified approach to enzymatic identification of osteoclastic bone resorption. J Histotechnol..

